# The T309G MDM2 Gene Polymorphism Is a Novel Risk Factor for Proliferative Vitreoretinopathy

**DOI:** 10.1371/journal.pone.0082283

**Published:** 2013-12-09

**Authors:** Salvador Pastor-Idoate, Irene Rodríguez-Hernández, Jimena Rojas, Itziar Fernández, María T. García-Gutiérrez, José M. Ruiz-Moreno, Amandio Rocha-Sousa, Yashin Ramkissoon, Steven Harsum, Robert E. MacLaren, David Charteris, Jan C. VanMeurs, Rogelio González-Sarmiento, José C. Pastor, on behalf of the Genetics on PVR Study Group

**Affiliations:** 1 Instituto de Oftalmobiología (IOBA-Retina Group), University of Valladolid, Valladolid, Spain; 2 Unidad de Medicina Molecular, Departamento de Medicina, University of Salamanca, Salamanca, Spain; 3 Instituto de Investigación Biomédica de Salamanca (IBSAL) and Instituto de Biología Molecular y Celular del Cáncer (IBMCC), University of Salamanca–CSIC-SACYL, Salamanca, Spain; 4 University of Castilla La Mancha, Vissum, Albacete, Spain; 5 Department of Sense Organs, Medical School, University of Porto, Hospital San João, Porto, Portugal; 6 Moorfields Eye Hospital, National Institute of Health Research (NIHR), Biomedical Research Centre, London, United Kingdom; 7 Nuffield Laboratory of Ophthalmology, University of Oxford, John Radcliffe Hospital Oxford, United Kingdom; 8 Rotterdam Eye Hospital, Erasmus Medical Center, University of Rotterdam, The Netherlands; University of Saarland Medical School, Germany

## Abstract

Proliferative vitreoretinopathy (PVR) is still the major cause of failure in retinal detachment (RD) surgery. It is believed that down-regulation in the p53 pathway could be an important key in PVR pathogenesis. The purpose was to evaluate the impact of T309G *MDM2* polymorphism (rs2279744) in PVR. Distribution of T309G *MDM2* genotypes among European subjects undergoing RD surgery was evaluated. Proportions of genotypes between subsamples from different countries were analyzed. Also, a genetic interaction between rs2279744 in *MDM2* and rs1042522 in *p53* gene was analyzed. Significant differences were observed comparing *MDM2* genotype frequencies at position 309 of intron 1 between cases (GG: 21.6%, TG: 54.5%, TT: 23.8%) and controls (GG: 7.3%, TG: 43.9%, TT: 48.7%). The proportions of genotypes between sub-samples from different countries showed a significant difference. Distribution of GG genotype revealed differences in Spain (35.1–53.0)/(22.6–32.9), Portugal (39.0–74.4)/(21.4–38.9), Netherlands (40.6–66.3)/(25.3–38.8) and UK (37.5–62.4)/(23.3–34.2). The OR of G carriers in the global sample was 5.9 (95% CI: 3.2 to 11.2). The OR of G carriers from Spain and Portugal was 5.4 (95% CI: 2.2–12.7), whereas in the UK and the Netherlands was 7.3 (95% CI: 2.8–19.1). Results indicate that the G allele of rs2279744 is associated with a higher risk of developing PVR in patients undergoing a RD surgery. Further studies are necessary to understand the role of this SNP in the development of PVR.

## Introduction

Proliferative vitreoretinopathy (PVR) is still the major cause of failure in retinal detachment (RD) surgery [1], affecting 5% to 10% of RD and accounting for approximately 75% of all primary failures after RD surgery [1], [2]. It is considered an abnormal wound-healing process induced by a retinal break allowing the posterior escape of retinal pigment epithelium cells (RPE) into a pro-inflammatory vitreous environment [3]–[7]. In any RD, the blood-ocular barrier breaks down, possibly due to disruption of the photoreceptor-RPE cell interface, then inflammatory cells are recruited increasing the inflammatory mediators production into the vitreous cavity [8]–[10]. Growth factors and cytokines in the vitreous cavity seem to be responsible for RPE migration, metaplasia and proliferation [11]–[13], which can result in the development of epi and subretinal membranes which are some of the characteristic clinical features of PVR. Tangential contraction of the membranes leads to reduced internal diameter of the retina and subsequent tension which rapidly develops into RD once a break allows ingress of subretinal fluid [14]–[16]. Some of those growth factors are also responsible of the glial cell hypertrophy causing important changes inside of the retinal tissue and inducing a shortening of the neuroretina, the most severe form of PVR [17].

Although PVR was identified in 1983 as an independent entity [18], and many efforts have been made for treating and preventing it during these years, there is neither current available medical treatment nor prophylaxis. Nowadays, treatment for PVR consisted of surgery, with an anatomically successful of 60% to 80% in the less severe cases and below of 40% in the most severe cases [19], [20]. Surgical procedures have a significant cost [21], involve the risk of recurrence [22], and above all have poor functional results [23]–[26]. Besides the efforts to identify nonsurgical approaches to treat PVR, they have not had success [27]–[29].

Most research has to date attempted to identify the clinical risk factors related to the development of PVR after RD; however, these clinical factors do not completely explain the probability of its onset [30]. Since PVR is a cell-based inflammatory response, like other inflammatory responses, genetic susceptibility may have an important role. In previous studies, we have partially described the contribution of the genetic component to PVR [31]–[34]. Single nucleotide polymorphisms (SNPs) have important implications for human genetic diseases and they may help to identify the genetic predisposition of certain diseases, either as a causative or protective factor.

Besides the role of many inflammatory mediators in the development of PVR and RD [35], previous studies have shown increased level of p53 and the activation of various cell death mechanisms after RD [36], [37]. Also it has been reported that photoreceptor death after RD and subsequent visual loss could be caused by apoptosis or other cell death pathways, such as programmed necrosis, when the pathways for apoptosis were inhibited by some drugs [38]–[40].

In normal unstressed cells, p53 is a very unstable protein with a half-life ranging from 5 to 30 min, which is present at very low levels owing to continuous degradation largely mediated by murine double min 2 protein (MDM2) [41], [42]. Importantly, MDM2 itself is the product of a p53-inducible gene [42]. Thus, the two molecules are linked to each other through an autoregulatory negative feedback loop aimed at maintaining low cellular p53 levels in the absence of stress and limiting the duration and severity of various p53-mediated biological responses after a non-lethal stress response. Conversely, a hallmark of many cellular stress pathways such as DNA damage, hypoxia, ischemia, telomere shortening, and oncogene activation is the rapid stabilization of p53 via a block of its degradation [41].

Recently, it has been reported that the levels of p53 expression could be a checkpoint in the development of RD and PVR, and how its local increase in the vitreous by using inhibitors of MDM2, seem to be a promising approach as a prophylaxis in experimental RD and also in experimental PVR [43].

The *MDM2* gene is a key negative regulator of p53 and in humans seems to have two promoter-enhancer regions that regulate the levels of *MDM2* mRNA. The first promoter is 5′ to the first exon and likely regulates the basal level of MDM2 in a nonstressed cell. The second promoter region is in the first intron and this region increases the expression of MDM2 after a p53 response [41], [42]. This intron is composed of 524 nucleotides with the T>G SNP at nucleotide 309. The G/G variant increases the binding affinity of the transcriptional activator Sp1 resulting in high levels of MDM2 protein; formation of transcriptionally inactive p53-MDM2 complexes and a consequent decreased activity of the p53 pathway [44], [45].

Thus, the purpose of this study was to analyze the distribution of the *MDM2* T309G polymorphism in the first intron of *MDM2* gene, in a consecutive sample of patients undergoing primary rhegmatogenous RD surgery with and without PVR, recruited from several European clinical centres through the project named Retina 4.

## Materials and Methods

### Ethics Statement

The study was approved by the local Ethics Committees of Instituto de Oftalmobiología (IOBA-Retina Group) (Valladolid, Spain), Hospital San João (Porto, Portugal), Moorfields Eye Hospital (London, United Kingdom) and Rotterdam Eye Hospital (Rotterdam, Netherlands) and followed the tenets of the Declaration of Helsinki. All patients received written informed consent before entering in the study.

### Design and study population

DNA samples from the Retina 4 project were analyzed. This work is made up in two steps: first, a candidate gene association study in the T309G polymorphism (rs2279744) located into the *MDM2* gene was carried out. Second, the interaction between this polymorphism and the *p53* codon 72 polymorphism (rs10425229) was investigated.

### Candidate gene association study

The association study was carried out among 555 patients from 7 centers: 3 in Spain, 2 in Portugal, 1 in the United Kingdom (UK) and 1 in Netherlands. The global sample was divided in sub-samples according to the country for the analysis. This study was carried out in two phases. In the first one, sub-samples from Spain and Portugal were analyzed. After significant results were found in this first cohort, subsequent samples from the UK and the Netherlands were analyzed (second phase). To compare if there were differences regarding geographical localization in the odds ratio analysis, Spain and Portugal were considered as Southern countries and the UK and the Netherlands as Northern countries. Genotypic and allelic frequencies were also compared between cases and controls in the global series.

Detailed explanation of the exclusion and inclusion criteria for classification of patients has been provided in a previous publication [33]. In brief, all participants were patients with a primary rhegmatogenous RD who underwent surgery. Exclusion criteria were: age under 16 years old; traumatic, tractional, exudative or iatrogenic RD; RD secondary to macular hole or giant retinal tears (larger than 3 clock hours) and pre-operative PVR grade higher than B. Those who did not develop clinical signs of PVR after 3 months of follow-up were included in the control group. Those who developed PVR grade C1 or higher**,** according to Machemer classification, were included as cases.

### Genetic interaction

Interaction between the *MDM2* polymorphism (rs2279744) and one SNP (rs1042522) located in the *p53* gene previously identified by our group as significantly associated to PVR [33] was investigated. Samples used for analyzing *p53* polymorphism were same samples than for the *MDM2* SNP. Carriers of Pro variant of rs1042522 and G variant of rs2279744, were analyzed in the global sample and in the sub-samples from different countries.

### Genotyping

Genotyping of the *MDM2* T309G polymorphism was performed at the Molecular Medicine Unit at the University of Salamanca, (Salamanca, Spain) blinded to the clinical status of patients, using the PCR-RFLP (Polymerase Chain Reaction-Restriction Fragment Length Polymorphism) technique [46], [47].

The *MDM2* T309G polymorphism was detected after amplification of genomic DNA with the forward primer 5'-GAGGTCTCCGCGGGAGTTC-3' and the reverse primer 5'- CTGCCCACTGAACCGGC-3' **.** *MDM2* intron 1 was amplified within a 155 pb DNA fragment that was digested with the restriction endonuclease MspA1I (New England Biolabs, Inc.).

PCR reactions were performed in a 50-µl reaction mixture containing 100–200 ng of target DNA, 20 pmol of each primer, 2.5 mM MgCl_2_, 50 µM of each dNTP and 1.25 U of HotMaster *Taq* DNA polymerase (5 Prime GmbH, Hamburg, Germany). DNA was amplified with the following steps: an initial 5-min denaturation at 94°C, followed by 35 cycles of 94°C for 30 s, 61°C for 30 s, 72°C for 30 s, and a final elongation at 72°C for 10 min.

**Figure 1:**
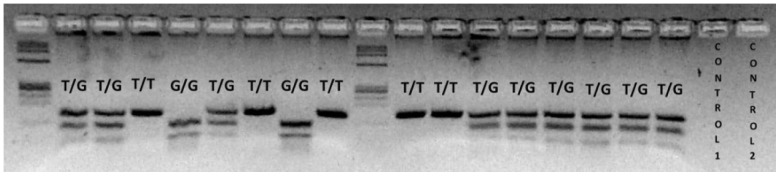
PCR-RFLP to determine *MDM*2 SNP309 polymorphism. *MDM*2 SNP309 T allele is not cleaved by MspA1I endonuclease and generates a single fragment of 155 bp. The *MDM*2 SNP309 G allele is cleaved by MspA1I and generates two small fragments of 101 and 54 bp. The *MDM*2 SNP309 heterozygote displays three fragments of 155, 101 and 54 bp.

The resulting fragments were separated on 3.5% agarose gel (Figure 1) and the ethidium bromide–stained fragments were analyzed under a UV source, using the Kodak Digital Science ID image analysis system.

The polymerase chain reaction fragments, containing T and G alleles, after digestion migrated as 2 fragments of 101 and 54 bp for G homozygotes (GG), 1 fragments of 155 for T homozygotes (TT), and 3 fragments of 157, 101 and 54 bp for heterozygotes (TG).

### Statistical analysis

The quality of data was evaluated in control sub-samples by Hardy-Weinberg equilibrium using the Chi-square test. Genotypic frequencies were estimated in each sub-sample. The proportions of genotypes and the G homozygote groups between sub-samples were analyzed. Also, the genotypic and allelic frequencies were compared between cases and control in the global sample and in the sub-samples from different countries. In the genetic interaction, patients carrying the Pro variant of rs1042522 and the G variant of rs2279744 were analyzed in the global sample and in the sub-samples from different countries.

Association was investigated using the Chi-Square and the Fisher’s tests. The strength of association was measured using Odds Ratio (OR) and 95% confidence intervals (CI). Two inheritance models were considered: co-dominant model that allows every genotype to give a different and non-additive risk, and recessive model in which two copies of the G allele are necessary to change the risk. The Akaike Information Criterion (AIC) [48] was used in order to choose the inheritance model that best fitted the data. The statistical analyses were performed by using SPSS 16.0 for Macintosh and R software (Software Foundation's GNU project) [49].

In order to adjust p-values for multiple comparisons a permutation test was performed. We used 1000 random shuffles of the case/control labels to get the correct distribution of test statistics under the no-association hypothesis. The ranking of the real test statistic among the shuffled test statistics gives the adjusted p-values.

## Results

### Candidate gene association study

A total of 555 peripheral DNA blood samples including 134 cases and 421 controls were analyzed (203 from Spain (36.57%), 68 from Portugal (12.25%), 121 from Netherlands (21.80%) and 163 from the UK (29.36%). Regarding clinical information some significant associations were observed. The control group was significantly older than cases (p<0.0001) with the difference between median of 6 years (95% CI: 3.39-8.31). A significant association in patients with history of PVR in the fellow eye was found in the cases group. Also status of the lens was determined because aphakia has been related to a higher incidence of PVR [5], [30] (Table 1). There were no significant associations with sex, race, affected eye or history of phakic status. There were no differences regarding the geographical localization or centre where the patients came from.

**Table 1:** Clinical characteristics of the whole sample.

Characteristics	Controls, n RD (%)	Cases, n PVR (%)	Total	P-value*
**Race**				0,064
-Caucasian	370 (73.56%)	133 (26.44%)	503	
-Hispano-American	6 (46.15%)	7 (53.85%)	13	
-Hindu	7 (58.33%)	5 (41.67%)	12	
-Arabic-North-African	6 (100%)	0	6	
-Sub-Saharan	2 (50%)	2 (50%)	4	
-Asian	3 (60%)	2 (40%)	5	
-Unknown			12	
**Sex**				0,362
-Male	248 (71.26%)	100 (25.37%)	348	
-Female	135 (75%)	45 (31.42%)	180	
-Unknown			27	
**Status of the lens (Phakia)**				0,233
-Yes	250 (74.63%)	85 (25.37%)	335	
-No	131 (68.58%)	59 (31.42%)	190	
-Unknown			30	
**RD in fellow eye**				0,528
-Yes	32 (76.19%)	10 (23.81%)	42	
-No	355 (68.58%)	136 (31.42%)	491	
-Unknown			22	
**PVR in fellow eye**				0,005(*)
-Yes	0	5 (100%)	5	
-No	141 (26.70%)	387 (73.30%)	528	
-Unknown			22	
**Geographical location**				0,171
-Northern countries (UK+Netherlands)	224 (78.87%)	60 (22.2%)	284	
-Southern countries (Spain+Portugal)	197 (72.7%)	74 (27.3%)	271	

RD: Retinal detachment; PVR: Proliferative vitreoretinopathy.

^*^ p-value: Chi-squared or Fisheŕs exact test in the statistical analysis of clinical characteristics of the whole sample of the global sample. (*) A significant association in patients with history of PVR in the fellow eye was found in the cases group.

There were no failures for the genotyping process, with a global call rate of 96.21%. Additionally, in order to ensure accuracy of allele-specific results, a randomized selection of samples PCRs were assessed by an independent researcher unaware of the patients’ status. All control sub-samples verified the Hardy-Weinberg equilibrium.

**Phase I**: genotypic distribution of *MDM2* T309G polymorphism in Spain and Portugal.

The frequencies of the genotypes in each country are shown in Table 2. The comparison of proportions of genotypes between sub-samples showed a significant difference (p<0.05) between cases and controls. Also a significant difference (p<0.05) in G homozygote carriers between sub-samples in the control group (CI G homozygote: Spain (22.6–32.9), Portugal (21.4–38.9)) and the cases group (CI G homozygote: Spain (35.1–53.0), Portugal (39.0–74.4)) was found.

**Table 2:** Distribution of genotypes and allelic frequencies in cases with PVR and controls.

Countries	Genotype	Controls	Cases	P-value Fisheŕs test	Corrected P-value	Alleles	Controls	Cases	Controls (95% CI Alleles)	Cases (95% CI Alleles)	P-value Chi Square test	OR*	CI OR 95%
**Spain**	T/T	72 (50.0%)	18 (30.5%)	0.0037^1^	0,0039^2^	G	80 (27.8%)	52 (44.0%)	(22.6–32.9)	(35.1–53.0)	0.0012^3^	2.0	(1.31–3.19)
	T/G	64 (44.4%)	30 (50.9%)			T	208 (72.2%)	66 (56.0%)	(67.0–77.4)	(46.9–64.9)			
	G/G	8 (5.6%)	11 (18.6%)										
**Portugal**	T/T	27 (51.0%)	3 (20.0%)	0.0387^1^	0,0449^2^	G	32 (30.2%)	17 (56.7%)	(21.4–38.9)	(39.0–74.4)	0.0156^3^	3.0	(1.31–6.95)
	T/G	20 (37.7%)	7 (46.7%)			T	74 (69.8%)	13 (43.3%)	(61.7–78.5)	(61.0–25.6)			
	G/G	6 (11.3%)	5 (33.3%)										
**United Kingdom**	T/T	66 (50.0%)	7 (22.6%)	0.0047^1^	0,0059^2^	G	76 (28.8%)	31 (50.0%)	(23.3–34.2)	(37.5–62.5)	0.0015^3^	2.4	(1.40–4.35)
	T/G	56 (42.4%)	17 (54.8%)			T	188 (71.2%)	31 (50.0%)	(65.7–76.6)	(37.5–62.5)			
	G/G	10 (7.6%)	7 (22.6%)										
**Netherlands**	T/T	40 (43.5%)	4 (13.8%)	0.0037^1^	0,0049^2^	G	59 (32.0%)	31 (53.5%)	(25.3–38.8)	(40.6–66.2)	0.0023^3^	2.4	(1.27–464)
	T/G	45 (48.9%)	19 (65.5%)			T	125 (68.0%)	27 (46.5%)	(61.1–74.8)	(46.5–59.3)			
	G/G	7 (7.6%)	6 (20.7%)										

^1^ Fisheŕs test. Ho. Independence between genotype case/control group. Significant differences were observed between cases and controls in the G/G genotype in Spain and Portugal, and in UK and Netherlands.

^2^ Permutation test for multiple comparison adjustment.

^3^ G homozygote carrier analysis between different countries revealed differences in Spain and Portugal as well as in Netherlands and UK.

^*^ OR : odds-ratio.

The odds ratio of G carriers from Spain and Portugal together considering a co-dominant model (T/T, T/G and G/G) (AIC =  307.7 vs 311.4 of a recessive model) was 5.4 (95% CI: 2.3 to 12.7) (Table 3).

**Table 3:** Models of inheritance in the global sample.

Model	Genotype	Controls	Cases	OR*	CI 95%	P-value	AIC*	Corrected P-value
**Co-dominant**	T/T	205 (48.7%)	32 (24.0%)	1.00		2.0374e-08	584.1	0,0009^1^
	T/G	185 (43.9%)	73 (54.4%)	2.53	(1.59–4.01)			
	G/G	31 (7.4%)	29 (21.6%)	5.99	(3.20–11.24)			
**Dominant**	T/T	205 (48.7%)	32 (24.0%)	1.00	-	2.1790e-07	590.7	0,0009^1^
	T/G-G/G	216 (51.3%)	102 (76.0%)	3.03	(1.95–4.70)			
**Recessive**	T/T-T/G	390 (92.6%)	105 (78.4%)	1.00	-	1.4218e-05	598.7	0,0009^1^
	G/G	31 (7.4%)	29 (21.6%)	3.47	(2.00–6,02)			
**Over-dominant**	T/T-G/G	236 (56.1%)	61 (45.6%)	1.00	-	3.3371e-02	613.0	0,0319^1^
	T/G	185 (43.9%)	73 (54.4%)	1.53	(1.03–2.26)			
**Spain+Portugal**	T/T	99 (50.3%)	21 (28.4%)	1.00	-	0.0003	307.7	0,0019^1^
	T/G	84 (42.6%)	37 (50.0%)	2.08	(1.13–3.82)			
	G/G	14 (7.1%)	16 (21.6%)	5.44	(2.30–12.7)			
**Netherlands+UK**	T/T	106 (47.3%)	11 (18.3%)	1.00	-	2.6566e-05	277.8	0,0009^1^
	T/G	101 (45.1%)	36 (60.0%)	3.43	(1.66–7.11)			
	G/G	17 (7.6%)	13 (21.7%)	7.30	(2.80–19.01)			

OR* (odds ratio).

AIC*(Akaike Information Criterion); The AIC is a measure of the relative goodness of fit of a statistical model. It can generally be used for the identification of an optimum model in a class of competing models.

Given a set of candidate models for the data, the preferred model is the one with the minimum AIC value.

^1^ Permutation test for multiple comparison adjustment.

Results of odds ratio using a co-dominant model for Spain plus Portugal and Netherlands plus United Kingdom (UK).

**Phase II**: genotypic distribution of *MDM2* T309G polymorphism in UK and the Netherlands.

The frequencies of the genotypes in patients from the UK and the Netherlands are shown in Table 1. Also, the distribution of genotypes between subjects from those countries showed statistical differences. When G homozygote carriers between cases and controls were analyzed a significant difference in both groups of patients was found in control (CI of G homozygote: Netherlands (25.3–38.8), UK (23.3–34.2)) and cases (CI of G homozygote: Netherlands (40.6–66.2), UK (25.3–38.8)). Also, differences were found in the odds ratio distribution of homozygous carriers of the G variant in patients from the UK and the Netherlands together considering a co-dominant model (AIC =  277.8 vs 288.4 of a recessive model) (odds ratio 7.3 (95% CI: 2.8 to 19.1) (Table 3).

When all samples were grouped, (Table 4) significant differences in the distribution of genotypes between the controls and cases (p<0.05) were found. Also homozygous carriers of the G variant were more frequent in PVR cases (CI: 42.9–54.8) than in controls (CI: 26.2–32.4). The odds ratio of the G variant in the global sample using a co-dominat model (AIC =  584.1 versus 598.7 of a recessive model) was 5.9 (CI: 3.2 to 11.2) (Table 3).

**Table 4:** Distribution of *MDM2* T309G polymorphism in the whole sample.

Genotypes	T/T	T/G	G/G	Total	CI 95%*	P-value Fisheŕs test	Corrected P-value	OR^2^	CI OR 95%^2^
**Cases**	32 (23.9%)	73 (54.5%)	29 (21.6%)*	134 (100%)	(42.9–54.8)*	1.6738e-08	0.0009^1^	2.3	(1.73–3.05)^2^
**Controls**	205 (48.7%)	185 (43.9%)	31 (7.4%)	421 (100%)	(26.2–32.4)*			-	-
**Total**	237	258	60	555					

1. Permutation test for multiple comparison adjustment.

2. OR. (Odds ratio).

^*^ Analysis of G homozygote carriers between case/control group.

Fisheŕs test.

### Allelic frequencies comparison

Significant differences in the analysis of the allelic frequencies were found between cases and controls in Spain and Portugal and between cases and controls in UK and Netherlands (Table 2)

### Genetic interaction

When the global sample was analyzed, 16 patients (2.91%) who meet both *p53* and *MDM2* genotypes (Pro variant plus G variant carriers) (8 patients from Spain and Portugal, and 8 patients from the UK and the Netherlands) were found. A significant difference in the distribution of genotypes between the controls and cases (p<0.0001) was found in the global sample. The odds ratio of Pro and G carriers in global sample was 10.19 (95% CI: 3.2 to 31.9) (Table 5).

**Table 5:** Genetic interaction between *p53* Pro72Arg SNP (rs1042522) and *MDM2* T309G SNP (rs2279744) in the global sample and in sub-samples from different countries.

Genotype	Sample	Controls	Cases	P-value	Corrected P-value	OR*	95% CI OR
**Pro/Pro + G/G**	Global	4	12	2.1139e-05	0.0009 1	10.19	(3.2–31.9)
	Spain+Portugal	1	7	0.0005	0,0009^1^	20.4	(2.4–169.5)
	Netherlands+UK	3	5	0.0130	0,0169^1^	6.5	(1.5–28.2)

^1^ Permutation test for multiple comparison adjustment.

OR*: odds ratio.

Results of patients (2.91%), who meet with both genotypes (Pro variant plus G variant carriers).

Significant differences in the distribution of genotypes between the controls and cases from Spain and Portugal together (p = 0.0006) and UK and Netherlands together (p<0.01) were found. The odds ratio of Pro and G carriers from the UK and the Netherlands together was 6.5 (95% CI: 1.5 to 28.2), whereas, the odds ratio of Pro and G carriers from Spain and Portugal together was 20.4 (95% CI: 2.4 to 169.5) (Table 5).

## Discussion

Our results show that Spanish and Portuguese carriers of the homozygous G SNP at position 309 have a 5.4 -fold increased risk of PVR after RD than those that carry the T allele. This observation was confirmed also in Dutch and British population (odds ratio 7.3 (95% CI: 2.8 to 19.1). Interestingly, when Pro and G restriction site carriers in the global sample were analyzed, an additional effect (odds ratio 10.19 (95% CI: 3.2 to 31.9) was found. But both polymorphisms were only present in 16 patients out of 555 patients. Although significant results were also found in the latter analysis, we consider that 16 may be too small a sample to draw absolute conclusions and further studies may be necessary in order to know the absolute potential risk of this restriction site association.

PVR is considered a multifactorial disease [31], [32] and may be the result of interaction between genetic and environmental factors [30]–[32]. The difficulty in identifying of patients at risk of developing PVR after RD by clinical characteristics [30] justifies our efforts to elucidate any genetic components [30]–[32]. A simple genetic test might identify higher risk patients before RD surgery, which might then be modified in a more personalized form.

Apoptosis is critically important during various developmental processes, it is necessary to rid the body of pathogen-invaded cells and also is involved in the removal of inflammatory cells and the evolution of granulation tissue into scar tissue [50]. Moreover, inappropriate apoptosis is an important factor in many human pathologic conditions including neurodegenerative diseases, ischemic damage, autoimmune disorders and many types of cancer. In addition, it has been reported that a deregulation of apoptosis during wound healing can lead to pathologic forms of healing such as excessive scarring and fibrosis [50].

As mentioned, PVR is considered an abnormal wound-healing process induced by the production of a retinal break and vitreal escape of RPE cells into an appropriate intraocular pro-inflammatory environment [3]–[7]. It is characterized by several intraretinal and extraretinal changes. One of the commonest is the extracellular matrix (ECM) formation, produced by activated-cytokines RPE cells (RPE cells transdifferentiate into mesenchymal like α-smooth muscle actin cells) in the vitreous cavity [51], [52]. This series of events culminates in the formation of a retina-associated membrane over and/or behind the neuroretina, which further contracts and thereby causes recurrent and tractional RD. Nevertheless the most severe changes are induced inside the retina by stimulating a reactive gliosis that causes a shortening of the retina preventing its surgical reattachment [17]. Some of these activating cytokines like transforming growth factor-β (TGFβ), and tumor necrosis factor α (TNFα), have been previously reported by our group in association with PVR [30]–[32].

Current studies have highlighted the involvement of extrinsic and intrinsic pathways of apoptosis in retinal cells after RD, and also, the existence of other pathways, such as programmed necrosis, when apoptosis are inhibited [40]. In addition, a proteomic study of human vitreous samples of RD and PVR has indicated that p53 could be involved in PVR process [53].

An association between *p53* Arg72Pro polymorphism and PVR has been recently reported by our group [33]. According to our results, carriers of the Pro allele of the *p53* gene, which are associated with a decrease in apoptotic function of p53, have higher risk of PVR after RD. Furthermore it has been reported that suppression of p53 expression might be a necessary event in the development of RD and PVR, and maintaining levels of p53 with agents such as Nutlin-3, which prevents the interaction between p53 and MDM2, might be effective in the prophylaxis of RD and also PVR vitreous-induced contraction [43].

The *MDM2* T309G polymorphism affects binding of transcription factor Sp1 and is associated with an increased expression of the MDM2 mRNA and attenuation of the p53 pathway [41], [42], [46], [47]. Interestingly, the elevated levels of MDM2 do not reduce the levels of p53 in non-stressed cells and the blockage of p53 binding to MDM2, using inhibitors of MDM2, promotes apoptosis [54].

Also, it has been reported that in contrast to other retinal cell types, the RPE cells are resistant to apoptosis, to TNFA and oxidative stress, which trigger apoptosis in wide range of cells [55], [56]. These factors, at normal range are generally ineffective in this cell type, however, it has also been reported that the use of inhibitors of MDM2 produces an increase of expression of proapoptotic targets capable of overcoming this inherent RPE resistance to apoptosis [54].

The *MDM2* T309G polymorphism has been associated to several cancers [44]–[47] and also to inflammatory processes [57]. Moreover, the association with other polymorphisms of *p53* (i.e. *p53* codon 72 polymorphism) increases the risk of development of several tumors [45], [46].

Results of this work suggest that carriers of the G allele of the *MDM2* gene, associated with a decrease in apoptotic function of p53, have higher risk of PVR after RD. We can speculate that the reduction in the levels of apoptosis in retinal cells may activates other cell death pathways, like programmed necrosis, which would increase the intraocular inflammation after RD, thus generating a cascade of tissue responses that generate and amplify the hostile microenvironment in which activated RPE can trans-differentiate. It is also possible that the decline in the levels of p53 is a crucial checkpoint in RD and PVR, and the recently developed ocular formulation of Nutlin-3, which can be administered by sub-conjunctival injection [58], could be an effective approach to achieve a prophylaxis in PVR disease.

This study had some limitations. One important issue in an association study is the sample size [59]. Probably, unlike other association studies, our sample is too small and the power sample is not enough to draw absolute conclusions, nevertheless, the sample collection to achieve greater power would be an extremely challenging for a low prevalence condition such as PVR. Cases were younger than controls and this could be considered a confounding factor. However, this difference was only of 6 year, which makes unlikely that this responds to a certain genetic profile. It is important to point out that functional polymorphisms are considered of interest because they allow us to shed light on the molecular basis of different pathologies. They also could be targets in the development of new therapeutic strategies. In this case-controlled study, we have identified one SNP within the *MDM2* gene that shows a strong association with PVR across several groups. Although we have carried out the study in two phases these findings must be interpreted with caution until these results are confirmed with further replication studies in order to confirm its association, because one of the major pitfalls of genetic association studies are the false positives [59], [60].

In summary, this study indicates that the p53 pathway could be implicated as a significant risk factor for PVR after RD and also it highlights the role of these SNPs (rs1042522 and rs2279744) as possible markers of PVR risk.
